# Sample phenotype clusters in high-density oligonucleotide microarray data sets are revealed using Isomap, a nonlinear algorithm

**DOI:** 10.1186/1471-2105-6-195

**Published:** 2005-08-02

**Authors:** Kevin Dawson, Raymond L Rodriguez, Wasyl Malyj

**Affiliations:** 1 Laboratory for High Performance Computing and Informatics and Section of Molecular and Cellular Biology University of California Davis MCB, One Shields Avenue Davis, CA 95616. USA

## Abstract

**Background:**

Life processes are determined by the organism's genetic profile and multiple environmental variables. However the interaction between these factors is inherently non-linear [1]. Microarray data is one representation of the nonlinear interactions among genes and genes and environmental factors. Still most microarray studies use linear methods for the interpretation of nonlinear data. In this study, we apply Isomap, a nonlinear method of dimensionality reduction, to analyze three independent large Affymetrix high-density oligonucleotide microarray data sets.

**Results:**

Isomap discovered low-dimensional structures embedded in the Affymetrix microarray data sets. These structures correspond to and help to interpret biological phenomena present in the data. This analysis provides examples of temporal, spatial, and functional processes revealed by the Isomap algorithm. In a spinal cord injury data set, Isomap discovers the three main modalities of the experiment – location and severity of the injury and the time elapsed after the injury. In a multiple tissue data set, Isomap discovers a low-dimensional structure that corresponds to anatomical locations of the source tissues. This model is capable of describing low- and high-resolution differences in the same model, such as kidney-*vs*.-brain and differences between the nuclei of the amygdala, respectively. In a high-throughput drug screening data set, Isomap discovers the monocytic and granulocytic differentiation of myeloid cells and maps several chemical compounds on the two-dimensional model.

**Conclusion:**

Visualization of Isomap models provides useful tools for exploratory analysis of microarray data sets. In most instances, Isomap models explain more of the variance present in the microarray data than PCA or MDS. Finally, Isomap is a promising new algorithm for class discovery and class prediction in high-density oligonucleotide data sets.

## Background

The gene expression microarray is an assay that measures expression levels of tens of thousands of genes in parallel on a single chip. Microarrays can be performed from a very small amount of a biological sample, thus allowing for an experimental design involving many sample groups, repeats, dense time series, and samples collected at high-granularity from various anatomic locations. Today, the cost of microarrays is the principal factor limiting the number of samples that can be examined in a particular experiment. In spite of the high cost of microarrays, two thirds of those surveyed by GenomeWeb said they performed more than 200 microarrays and 57% spent more than $100,000 on microarrays in 2003 [2]. Sixty eight percent of these chips were oligonucleotide arrays, mostly Affymetrix chips. With the widespread use of microarrays in basic research and their increasing use in medical diagnostics, biomedical researchers can anticipate lower costs for chips that will lead to more studies utilizing hundreds, if not thousands, of samples. This expansion in sample size will provide researchers with higher resolution insights into biological processes as they are reflected in temporal, spatial, and functional patterns in microarray data sets. To reveal these patterns, several types of pattern recognition and clustering techniques have been developed and applied to microarray data.

A common task in the analysis of large microarray data sets is sample classification based on gene expression patterns. This process can be divided into two steps: class prediction and class discovery. During class prediction samples are assigned to predefined sample classes; whereas class discovery is the process of establishing new sample classes. For example, when gene expression arrays are used for cancer classification, class prediction assigns tumor samples into pre-existing groups of malignancies, while class discovery reveals previously unknown cancer subtypes [3]. The newly discovered tumor subtypes may have different clinical patterns, respond differently to certain drugs, and require more or less aggressive surgical and radiological treatment. Class discovery may also reveal previously unknown processes in cancer biology and define more specific indications for certain drugs. Specific drugs may be used to target newly discovered tumor subtypes, thus facilitating pharmacogenomic drug design and development. These goals will soon become achievable with the results from microarray studies using large samples. Class prediction and class discovery using large data sets will require the evaluation, adaptation, and development of robust mathematical, statistical, and computational tools.

Several mathematical algorithms and computational methods have been applied to class prediction and class discovery in large gene expression data sets. The methods most frequently used are based on clustering techniques such as hierarchical clustering (HC) [4]. HC was used for temporal classification in conjunction with Fourier analysis to detect genes that correlate with periodic changes in synchronized *S. cerevisiae *cells [5]. HC was also applied to cancer classification, for example breast cancer classification [6]. Other clustering techniques applied to microarray data are the unstructured *k*-means clustering [7], cluster affinity search (CAST) [8], fuzzy c-means clustering [9], and two-way clustering [10] that was used for the analysis of drug-tumor interactions [11]. Self-organizing maps (SOM) is another technique that is particularly well suited for exploratory data analysis. Unlike HC, SOM does not impose a rigid structure to the data [12]. The utility of SOM was demonstrated in leukemia classification using a weighted voting procedure [3]. Weighted voting classification was also used for predicting chemosensitivity of the NCI-60 tumor collection [13] and human breast cancers [6]. Supervised methods, such as Fisher's linear discriminant analysis, artificial neural networks (ANN), support vector machines (SVM), and boosting, have the advantage that the sample classes are usually defined by another "gold standard" method (e.g., histology, clinical outcome, length of survival, etc). In supervised classification, the choice of classifiers is frequently based on other considerations, e.g., genes that play a role in the pathomechanism of a certain disease or are expressed in a particular tissue. These "enrichment" methods can improve the prediction strength of a classification and decrease the sample number necessary for developing a prediction model. However they also introduce bias that may lead to overtraining or lack of discovery of unexpected sample classes. One of the supervised methods, support vector machines (SVM), has the advantage that it does not make assumptions about the distribution of the data [14]. SVM was tested on ovarian cancer, leukemia, and colon tumor data sets [15] and was also demonstrated to be useful for multi-class cancer classification [16-18]. Artificial neural networks (ANN), another machine learning method, was shown to classify small, round blue-cell tumors (SRBCT) [19]. Tree harvesting, a new method of supervised learning was recently applied for gene expression data [20]. In contrast to these complex procedures, much simpler classifiers may also perform equally well on some data sets. For example, nearest shrunken centroids were applied to SRBCT cells and leukemias [21].

Dimensionality reduction methods are useful in predicting the underlying true dimensionality of a microarray data set and reduce the number of variables applied as inputs to any of the classification procedures. Multi-dimensional scaling (MDS), a linear method, was used for classifying alveolar rhabdomyosarcomas [22], cutaneous malignant melanomas [23], and breast cancers [24]. Principal component analysis (PCA), another dimensionality reduction method, was used for visualizing gene expression maps of central nervous system (CNS) development [25] and classifying embryonal CNS tumors [26]. Probabilistic PCA, a method incorporating biological assumptions in linear factor models, was recently applied to two yeast microarray datasets [27]. Another method, singular value decomposition (SVD), was applied to soft tissue tumors [28], *S. cerevisiae *cell cycle, and serum-induced fibroblast data sets [29,30]. Generalized SVD (GSVD) was developed to extract conserved gene expression patterns comparable between two different organisms [31]. All these linear methods are inherently sensitive to outliers, missing values, and non-normal distribution. A variant of SVD, robust SVD (rSVD), was recently developed to minimize the effect of these corruptions in the data set [32]. Sammon mapping, a nonlinear mapping algorithm [33] was incorporated in the R *multiv *package as well as gene expression data processing and exploratory analysis software, such as ENGENE [34]. To address similar issues, Isomap [35] a nonlinear technique of dimensionality reduction originally designed for solving classical problems of pattern recognition, such as visual perception and handwriting recognition, was applied recently to discovery biologically relevant structures in cDNA microarrays [36,37].

We have applied Isomap to the analysis of both breast cancer microarray data sets and prostate cancer proteomics spectra [36] and showed that it consistently outperformed PCA in revealing biologically relevant low-dimensional structures in high-dimensional data sets. Nilsson *et al*. independently demonstrated the utility of Isomap using a lymphoma and a lung adenocarcinoma cDNA microarray data set [37]. We report here the application of Isomap to three independent Affymetrix GeneChip^® ^data sets. We show that Isomap is capable of discovering temporally, spatially, and functionally relevant structures in gene expression data. *To avoid any kind of bias that may be introduced with gene selection or feature enrichment methods, we did not apply any data scrubbing, filtering, or feature enrichment techniques*. We also show that Isomap can successfully detect biologically relevant structures even in the background noise of tens of thousands of genes present.

## Results

### Spinal cord injury data set

The Isomap algorithm was first evaluated on a large data set consisting of 170 rat U34A high density oligonucleotide arrays with 8,799 genes on each array [38]. These data were originally collected for a study on spinal cord injury that illustrated the role of cell cycle in trauma-induced neuronal death [38]. Compared to earlier microarray studies on experimental spinal cord damage, this study applied a lower level spinal cord injury, used individual rat samples rather than pooled spinal cord tissues, evaluated several time points, employed larger Affymetrix arrays, and used both sham-injured and naïve controls.

Unlike this original study, we do not make the assumption that only those genes "consistently expressed above background" are important for further consideration. The DiGiovanni *et al*. study used a stringent inclusion threshold that included only those genes present in at least 40% of all the samples. In addition to this first filter, a second filter was also applied that eliminated genes that did not have a change in expression level of at least two-fold compared to that of the sham controls, which was determined with Welch ANOVA *t*-test. Unlike the DiGiovanni *et al*. study, our analysis considers all 8,799 genes, thus eliminating an important source of potential selection bias. We do not use stringent selection criteria at the level of data scrubbing, because these filters may introduce confounding into the data set, which can lead to separation between sample classes based on subjective filtering criteria rather than existing biological phenomena.

**Figure 2:**
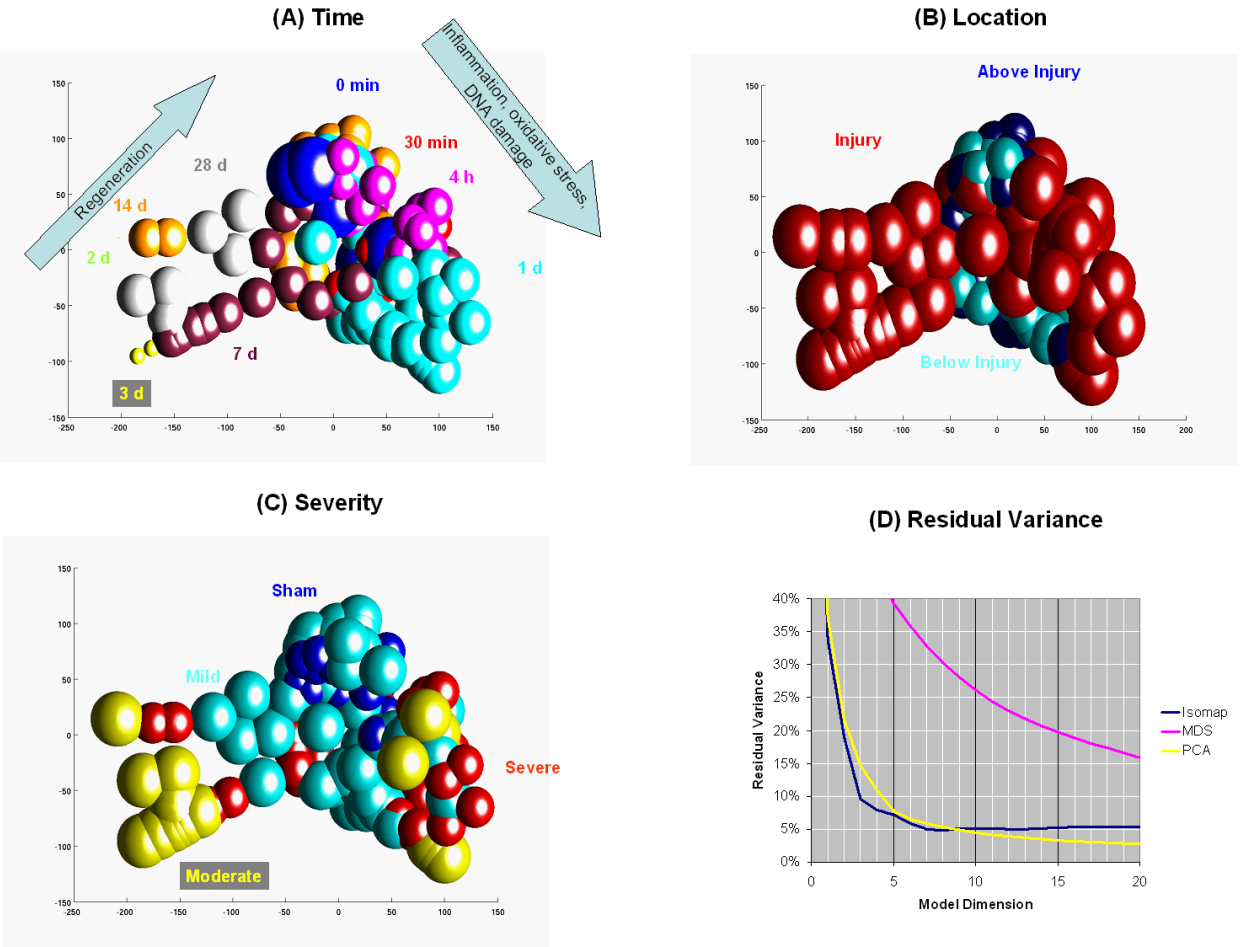
**Isomap analysis of the spinal cord injury data set. **Three-dimensional Isomap models were generated from 170 rat high density oligonucleotide arrays with 8,799 genes on each array as described in *Systems and Methods*. Samples were classified based on the ***A*: ***time*, ***B*:***location*, and ***C*:***severity *of the spinal cord injury. Sphere diameters express the 95 percentile distance from the nearest neighbor within the group. Neighborhood size: *k *= 3. ***D*: **Residual variance after the application of Isomap, MDS, and PCA models. See text for more detail. Animated images are presented in Additional Files 1, 2, 3.

One hundred seventy samples were collected from spinal cords of naïve rats, after sham operations, as well as mild, moderate, and severe injuries. In addition to these five severity classes, samples are also classified into one of three locations, such as below, above, and at the position of the spinal cord injury. The third classification category is the time interval from the injury to the sample collection. These time points are: 0 min, 30 min, 4 h, 24 h, 2 days, 3 days, 7 days, 14 days, and 28 days. All the 170 samples were subjected to Isomap analysis in a completely unsupervised fashion without the *a priori *knowledge of which classes the sample belongs to. Isomap fits a nonlinear manifold on the 170 samples. This manifold is used to express sample-to-sample distances as path distances on the surface of the manifold rather than the direct Euclidean distance without considering the existence of the manifold. Distances are computed for all pairs of the 170 samples and subjected to multi-dimensional scaling. The result of this procedure is presented in Figure 2 in a three dimensional coordinate system where each sphere represents one sample.

Figure 2 shows the 170 samples in a three-dimensional model that was generated by Isomap. Samples are classified and colored according to one of three major attributes – time, location, and severity of the injury. The sphere diameters express the compactness of the sample classes; the more compact the class, the smaller the diameter of the sphere. If members of a class spread out in a larger space then larger size spheres are displayed. The sphere diameter is determined as the 95 percentile of the distance distribution from the nearest neighbor within the class. The Isomap model successfully clusters similar samples into groups that are at well-defined locations of the three-dimensional model. The least affected sham-operated samples are shown at the very central core location of the model. By contrast, the most affected samples after moderate to severe injury at 24 h (that is, the most active phase of spinal cord injury) are shown at the peripheries of the model.

The *location *panel (Fig. 2A) shows that samples from regions below and above the injury are at the central core of the model. At the same time, samples from the injury itself are displayed in the peripheral lobes of the model. There is no visible separation between the unaffected samples and those originating from locations other than the injury itself.

The *time *panel (Fig. 2B) shows a clear-cut time-dependent separation of the samples along the *x*-axis. The right-side lobe of the model shows samples at 30 min, 4 h, and 24 h after injury. The earlier samples are at the core of the model. Conversely, samples from later time points are found at more distal locations in the right-side lobe of the model. This time-pattern is in agreement with Di Giovanni *et al*. [38] who found with temporal clustering that the immediate early genes are over-expressed at 30 min after spinal cord injury. This is followed by genes associated with inflammatory and oxidative stress plateauing at 4 h after injury, as well as cell cycle and neuronal apoptosis-regulating genes at 4–24 h. Isomap effectively separates this early active phase of post-injury damage from the later phase of regeneration that takes place at 48 h – 28 days. Samples from this later regenerative phase, at time points of 2, 3, 7, 14, and 28 days, are displayed in the left-side lobe of the Isomap model. During regeneration, the earlier samples are shown in the more peripheral locations and the later samples are closer to the central core of the model. It is noteworthy that some samples are at the peripheral locations of the model even 28 days after the injury, which is explained by incomplete regeneration and permanent damage caused by a more severe spinal cord injury as will be demonstrated later. In the *time *panel, Isomap shows the most striking separation between the early and later phases of post-injury events. The right-side lobe of the model contains samples from the early 30 min – 24 h phase of the post-injury damage. In these samples, the dominant events are the spinal cord injury, the secondary biochemical changes, and the endogenous autodestructive events. On the other hand, the left-side lobe of the Isomap model contains samples from the later 48 h-28 day phase when the neuroprotective and recovery promoting phenomena overcome the earlier autodestructive events.

The *severity *panel (Fig. 2C) shows that samples taken after incremental levels of spinal cord injury appear at increasingly distal locations in the Isomap model. The sham-operated samples are located in the central core of the model surrounded by a shell of samples from rats with mild spinal cord injury. The most peripheral samples in both lobes of the model are those representing moderate to severe injury. In the right lobe of the model, which represents the 30 min – 24 h time points, there is no clear separation between moderate injury samples and those with severe injury. Unexpectedly, in the left lobe that represents the later time points, the samples with moderate injury are more distal than those with severe injury. Although this separation between the moderate and severe injury was not anticipated, the separation between mild and moderate to severe injury is very clear. This separation also answers a question we raised on the *time *panel. In the left lobe of the model, six samples are visible at 28 days after injury. Three of these samples are in a cluster that is closer to the periphery and the other three samples are closer to the core of the model. The *severity *panel clearly shows that the three distal samples are those that underwent moderate injury and the more central three samples are those with only mild injury. This finding is consistent with our interpretation that the left lobe of the model represents the regeneration process. While three rats were able to partially recover from a mild injury after 28 days, the other three animals with moderate spinal cord injury did not recover by this time.

**Figure 1:**
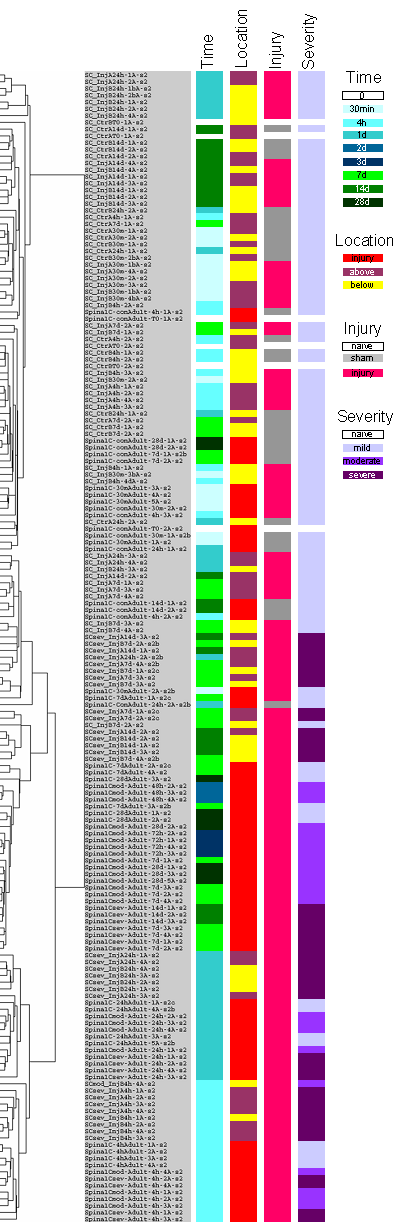
**Hierarchical clustering of samples in the spinal cord injury data set. **One hundred seventy rat high density oligonucleotide arrays were clustered with hierarchical clustering as described in *Systems and Methods*. The samples are annotated on time, location, and severity of the injury.

For comparison, in addition to Isomap analysis we also used hierarchical clustering [4] to cluster the 170 samples based on their gene expression patterns (Fig. 1). After clustering, the orientation of the branches of the clustering tree is undefined. Therefore, we used SOM to fold the leaves in an order that places the more similar samples closer to each other [12]. Although hierarchical clustering and SOM clusters similar samples into well-defined groups, the result is inferior to the Isomap model because the tree structure can display only one-dimensional structures, which is inadequate for displaying complex multi-dimensional phenomena such as spinal cord injury. In this experiment, several modalities are present, such as location and severity of the injury and the elapsed time after injury. Unlike clustering, Isomap analysis discovers these three major modalities and presents the 170 samples in a fashion that the underlying biological processes can be interpreted based on these three modalities.

### Rat multiple tissue gene expression data set

Isomap was applied to another data set containing 122 samples from 11 peripheral tissues and 15 brain regions from three common outbred rat strains, such as Wistar, Wistar Kyoto, and Sprague Dawley. The original study was performed to characterize physiological expression levels in these anatomical locations, to find genes that are important in certain brain regions, and to explain the phenotypic variations between rat strains [39]. This data set consists of 122 Affymetrix rat U34A arrays with 8,799 genes on each array.

Using Affymetrix MAS5, Walker *et al*. applied several filters on the genes, such as *t*-test p-value less than 0.05, at least two-fold change of expression levels, and minimum expression thresholds. Additionally, they used Rosetta Resolver with two filters such as Resolver ANOVA p-value less than 0.05 and at least two-fold change of expression levels. Our Isomap analysis of this data set used all genes to compute sample-to-sample differences without the use of input filters. Although our approach may increase the noise in the data set, it also eliminates any potential source of selection bias. We also avoided using any tissue-enrichment techniques based on *a priori *knowledge. Isomap analysis was carried out in a completely unsupervised fashion.

We were surprised by the ability of Isomap to sort several rat tissue samples in a fashion that reflects the topological anatomy of the source tissues (Fig. 2A). It is apparent in this figure that duplicate samples from the same tissue are displayed at almost identical locations on the Isomap map. For example, on the top left side of the figure, duplicates from small intestine, large intestine, and endothelial samples are presented as three dots. These three tissues are displayed close to each other, which is expected since the primary components of all three are epithelial cells. Nearby is displayed the kidney, a large part of which is also derived from epithelial cells. Below these samples on the mid left part of the figure close to each other are the spleen and thymus samples, two organs of the immune system. On their right is the bone marrow which is the primary site of blood cell generation and therefore functionally related to the spleen and thymus. On the bottom left part of the figure, close to each other are the two major muscle tissues, such as the skeletal muscle and the heart muscle. At the bottom of the figure is the cornea that is an ectodermal tissue close to the brain samples that are also ectodermal in origin and farther away from the endodermal and mesodermal tissues mentioned above. The two endocrine glands of the brain, such as the pineal and pituitary glands, are somewhat separate from the other parts of the brain.

**Figure 3:**
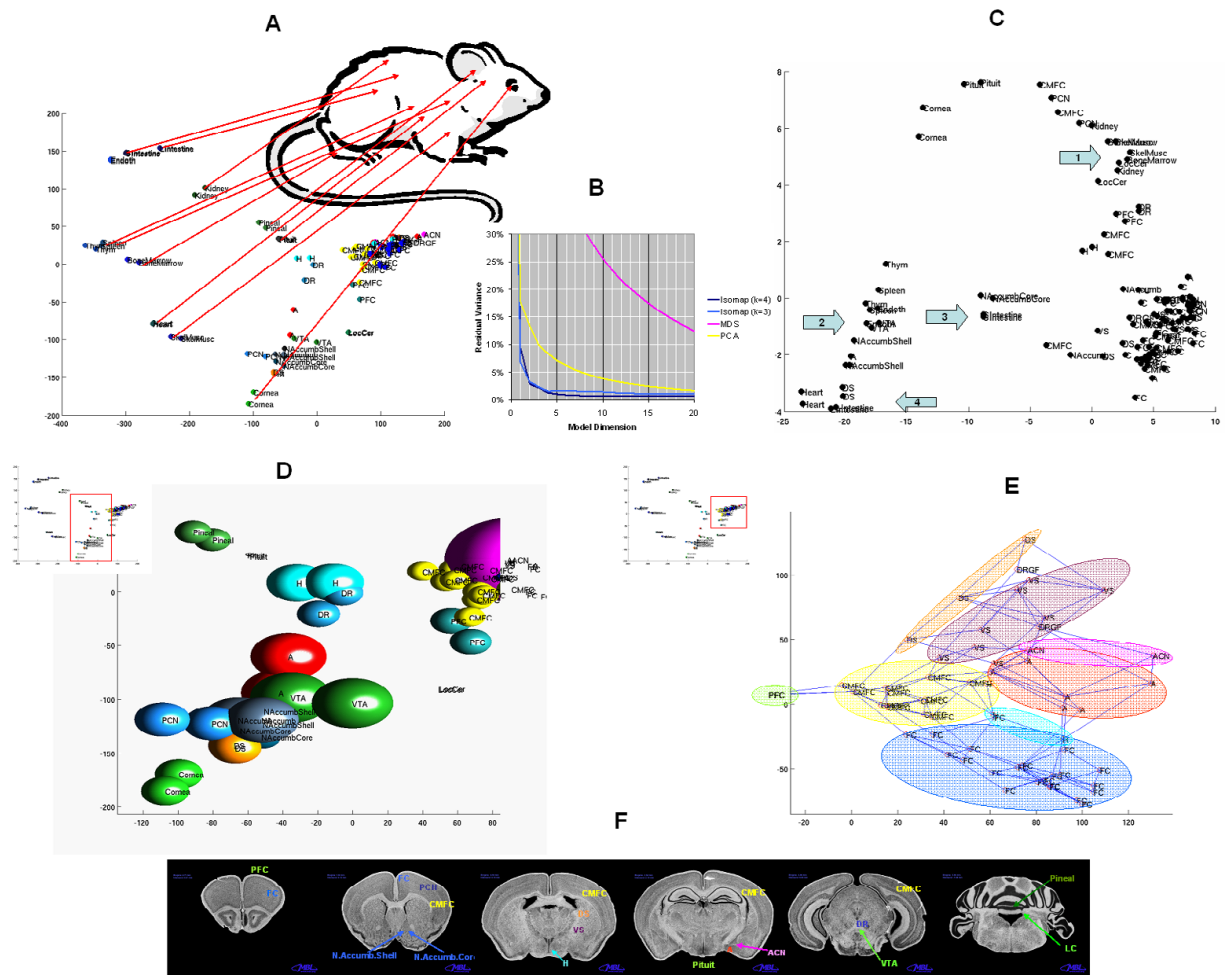
**Isomap analysis of the rat multiple tissue gene expression data set. **Three- and two-dimensional Isomap models were generated from a data set containing 122 samples from 11 peripheral tissues, 15 brain regions of three common out-bred rat strains, such as Wistar, Wistar Kyoto, and Sprague Dawley as described in *Systems and Methods*. Panel ***A ***shows an overview of a two-dimensional model using *k *= 3 nearest neighbors. Panel ***B ***shows the residual variance after the application of Isomap, MDS, and PCA models. Panel ***C ***shows a 2-dimensional comparison map generated by Principal Component Analysis. This panel demonstrates that PCA, unlike Isomap, is less accurate in mapping the different tissues correctly. Arrows show that (1) the skeletal muscle and kidney samples are overlapping the forebrain samples, (2) the thymus and spleen samples overlap the nucleus accumbens samples, and (3) the small intestine samples are mapped at a distance from the (4) large intestine samples. Panel ***D ***shows a portion of panel *A *with a three-dimensional model with *k *= 4. Panel ***E ***presents another region of the overview map representing the central brain structures. The neighborhood graphs demonstrate the *k *= 3 nearest neighbors. Panel ***F ***presents the anatomic locations of the samples on brain slice images from the Neuroscience Division, Regional Primate Research Center, University of Washington: BrainInfo (2000) . The abbreviations are: *A*, amygdala; *ACN*, amygdala central nucleus; *CMFC*, cortex minus frontal cortex; *DR*, dorsal raphe; *DRGF*, Fisher dorsal root ganglia; *DS*, dorsal striatum; *endoth*, endothel; *FC*, frontal cortex; *H*, hypothalamus; *LIntestine*, large intestine; *LocCer*, locus ceruleus; *NAccumb*, nucleus accumbens; *PCN*, primary cortical neurons; *PFC*, prefrontal cortex; *Pineal*, pineal gland; *Pituit*, pituitary gland; *SIntestine*, small intestine; *SkelMusc*, skeletal muscle; *Thym*, thymus; *VS*, ventral striatum; and *VTA*, ventral tegmental area. See text for more detail.

Figure 3D is a three-dimensional Isomap model of an enlarged region of panel *A*. The bottom left corner of this figure is the cornea below the primary cortical neurons (PCN). Right of the PCN is the dorsal striatum (DS) and the nucleus accumbens. Walker *et al*. were specifically interested in studying drug-seeking behavior; therefore separate samples were collected from the core and shell regions of the nucleus accumbens. In our Isomap model, these samples are displayed very close to each other. Above the nucleus accumbens is displayed the ventral tegmental area (VTA) and the amygdala (A). Near to the top of the figure are the dorsal raphe (DR) and the hypothalamus (H). Left of the hypothalamus is the pituitary, an endocrine gland under the control of the hypothalamus. Even farther left is the pineal gland, another endocrine organ related to the pituitary gland. On the right middle part of panel *D*, are two samples from the locus ceruleus presented at almost identical locations in the Isomap model. In the top right portion of panel *D *are cortical locations that are displayed in panel *E *in a two-dimensional Isomap model.

Figure 3E is a two-dimensional Isomap model of another enlarged region of panel *A*. This panel shows the neighborhood graphs used for building the Isomap model. On the left side of this panel is represented the prefrontal cortex (PFC) that is connected to the temporal/parietal/occipital cortex (cortex minus frontal cortex, CMFC). At the bottom of this panel are the samples from the frontal cortex (FC) below two hypothalamus (H) samples. Above these are samples from the amygdala (A), the amygdala central nucleus (ACN), which is a part of the amygdala, the ventral striatum (VS), and the dorsal striatum (DS). Not circled are two samples from the Fisher dorsal root ganglia (DRGF). Figure 3E also shows that most of the amygdala samples are close to the ACN. Unexpectedly, two other amygdala samples are in a very different location showed on panel *D *close to the VTA. Similar to the amygdala, the hypothalamus (H) is also shown at two different locations; two hypothalamus samples are shown in panel *E *below the amygdala, and two other samples are in panel *D *close to the dorsal raphe (DR).

Isomap analysis projects the hypothalamic samples onto two different well-defined locations in the Isomap model. This separation of the hypothalamus samples may reflect an unequal contribution of the different hypothalamic regions to the four hypothalamic samples and provides an example of class discovery. The hypothalamus has several anatomic regions, one of which is the periventricular hypothalamus that is functionally related to the pituitary as well as to the autonomic areas in the brain stem and the spinal cord. Another hypothalamic region, the medial hypothalamus has several connections with the medial division of the amygdala. The third hypothalamic region, the lateral preoptic hypothalamus, has a very complex anatomy with many fibers passing through this region. These subclasses may be explained by spatial or temporal differences. The hypothalamus samples on panel *D *close to the pituitary samples may originate from samples that are rich in periventricular hypothalamus. On the other hand, samples on panel *E *close to the amygdala samples may be richer in medial hypothalamus. The separation of the hypothalamus samples into two well defined classes may be explained by not only anatomical but temporal differences. Many of the hypothalamic nuclei synthesize several neurotransmitters and hormones, such as corticotropin-releasing hormone (CRH) and other related releasing hormones. The levels of these molecules and the activity of particular hypothalamic neurons may vary by time depending on the circadian rhythm, stress, food intake, emotional state, estrus cycle, and other factors. The two hypothalamic subclasses discovered by Isomap may be different because of these temporal variations.

Most of the amygdala (A) samples are projected on the Isomap map at a location between the cortical regions and the ventral striatum (Fig. 3E). Two samples, on the other hand, are projected at the ventral tegmental area (VTA) and the nucleus accumbens (panel *E*). These two amygdala samples are clearly different from the rest of the amygdala samples. This separation is consistent with anatomical dissimilarities between different parts of the amygdala. The largest portion of the amygdala, the basolateral nuclear complex, primarily consists of pyramidal and stellate neurons similar to those in the cerebral cortex. In fact, Isomap found most of the amygdala samples in the proximity of the cerebral cortex. Another part of the amygdala, the centromedial group, including the central nucleus (ACN), is connected to the bed nucleus through fibers called the stria terminalis. Although the bed nucleus is anatomically closer to the hypothalamus, it is histologically very similar to the amygdala. This separation demonstrates a potential class discovery by Isomap, where the discovered classes are in agreement with anatomically, histologically, and functionally well defined structures within the hypothalamus and the amygdala. Some of the hypothalamus and amygdala samples potentially contain more or less tissue from different parts of these anatomically heterogeneous brain structures, which leads to different classifications based on the major constituent.

As a comparison, we analyzed the multiple tissue dataset also with Principal Component Analysis (PCA) (Fig. 3C). Unlike Isomap, PCA was unable to separate the skeletal muscle, kidney, and bone marrow samples from each other (arrow 1). With PCA, the thymus and spleen samples overlap the nucleus accumbens samples (arrow 2); and the small intestine (arrow 3) and large intestine (arrow4) samples are mapped at a distance from each other. This comparison demonstrates that Isomap outperforms PCA in mapping the different tissue samples on a 2-dimensional space. This difference in performance of the two methods is also supported by the higher residual variance of the PCA model compared to that of the Isomap model (Fig. 3B).

### High throughput drug screening data set

The Isomap algorithm was also applied to a high throughput drug screening data set [40]. The goal of this study was to develop a general approach for identifying gene expression signatures as surrogates for cellular states in high throughput drug screening experiments. Particularly, Stegmaier *et al*. screened 1,739 chemical compounds to identify those capable of inducing terminal differentiation of acute myeloid leukemia (AML) cells. They exposed undifferentiated HL-60 samples (undiff) to several chemical compounds. The names and abbreviations of the chemical compounds selected for microarray analysis are listed in Table 1. In addition to the HL-60 samples, Stegmaier *et al*. also included primary acute promyelocytic leukemia (APL), primary patient AML, normal human neutrophil (poly), and normal human monocyte (mono) cells (Table 2). This gene expression data set consists of 86 human genomic U133A Affymetrix arrays with 18,400 transcripts and variants on each array and 30 human 6800 arrays with 7,129 genes on each array.

**Table 1:** Chemical compounds and their abbreviations used in the high throughput screening dataset

Apo	(R)-(-)-apomorphine HCl
ATRA	all *trans *retinoic acid
Caff	8-(3-chlorostyryl) caffeine
Cyc7p5	cyclazosin HCl
DMSO	dimethyl sulfoxide
EGFR	4,5-dianilinophthalimide
Erythro	erythro-9-(2-hydroxy-3-nonyl)adenine HCl
Keto	16-ketoestradiol
Methyl	α-methyl-L-p-tyrosine
Perg	pergolide methanesulfonate
Phen	1,10-phenanthroline
PMA	phorbol-12-myristate-13-acetate
Scop	(-) scopolamine methyl bromide
Sulma	sulmazole
VitD	calcitriol
5FU	5-fluorouracil
5FUD	5-fluorouridine

**Table 2:** Cells and their abbreviations included in the high throughput screening dataset

AML	primary patient acute myeloid leukemia cells
APL	primary acute promyelocytic leukemia cells
undiff	HL-60 cell line
mono	normal human monocytes
poly	normal human neutrophils

**Figure 4:**
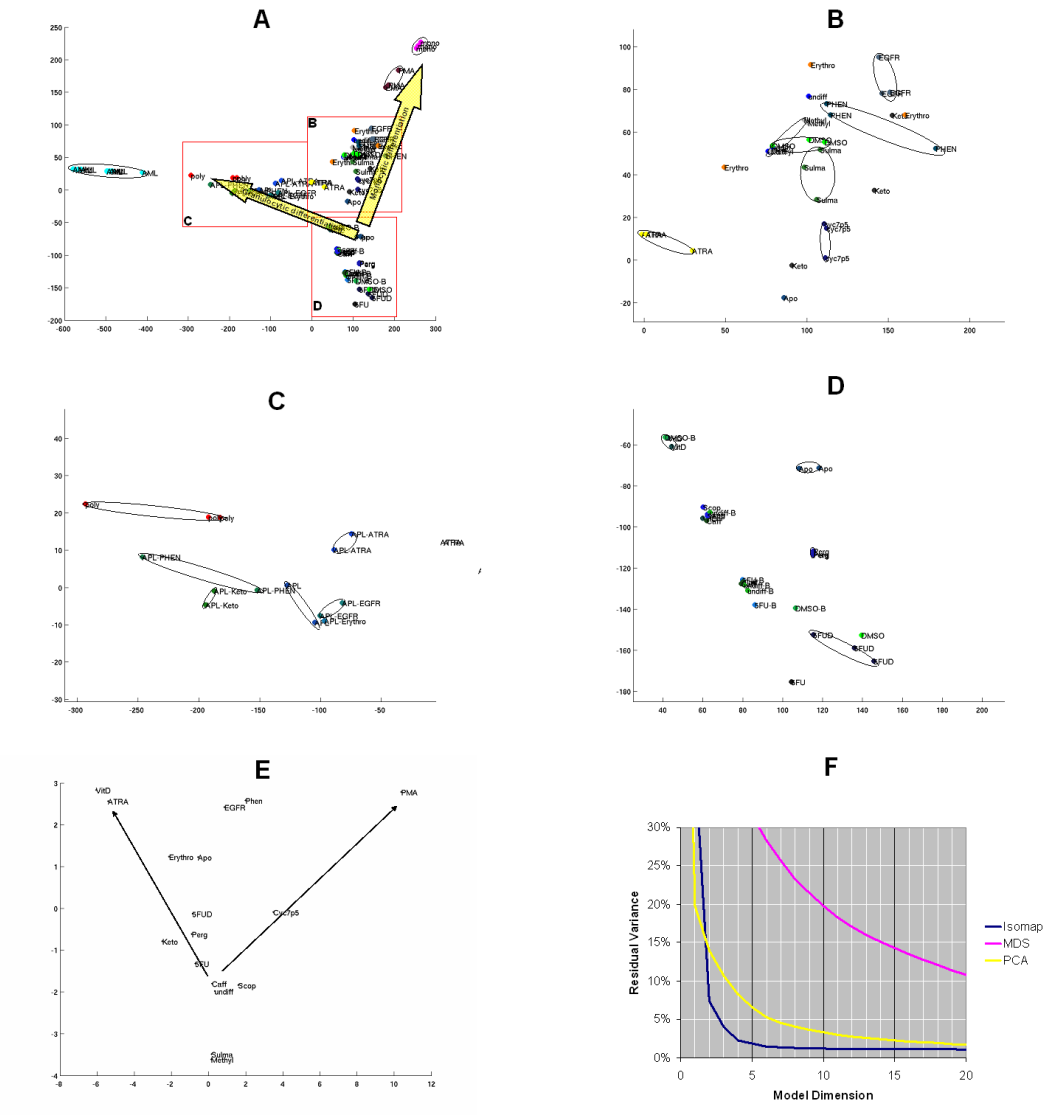
**Isomap analysis of the high throughput screening data set U133A arrays.**Two-dimensional Isomap models were generated from 86 human U133A high density oligonucleotide arrays with 22,283 genes on each array as described in *Systems and Methods*. Panel ***A ***shows an overview map of the Isomap model. Arrows point to the directions of the monocytic and granulocytic differentiation. Panels ***B, C*,**and ***D ***are zoomed in regions of panel *A*. Panel ***E ***shows Isomap analysis of the monocytic and neutrophilic differentiation markers as described in *Systems and Methods*. Neighborhood size *k*: = 4. Panel ***F***shows the residual variance after the application of Isomap, MDS, and PCA models. Abbreviations of compounds and cell types are listed in Table 1 and 2, respectively. See text for more detail.

Figure 4 shows a two dimensional Isomap model built from 86 human U133A microarrays. Panel *A *presents a low resolution overview of this model. The 86 samples are distributed in a **Y**-shape with the untreated patient AML samples and normal neutrophils clustered at one end of the **Y **and the normal monocytes at the other. The two perpendicular arrows on this panel represent the granulocytic and monocytic differentiation. The former arrow points from the HL-60 cells (undiff) to the normal neutrophils (poly) and the latter one points to the normal monocytes (mono). As expected, HL-60 cells treated with *PMA*, a well characterized inducer of monocytic differentiation, are clustered close to the normal monocytes. It is noteworthy that the gene expression patterns of the primary patient AML cells are very different from that of the HL-60 cell line.

Figure 4B, an enlarged portion of panel *A*, focuses on the monocytic end of the **Y **distribution showing HL-60 cells treated with several chemical compounds. These compounds, *Apo*, *Keto*, *Cyc7p5*, *Sulma*, *Erythro*, *DMSO*, *Methyl*, *Phen*, *EGFR*, and *PMA *(Table 1) change the gene expression pattern of HL-60 cells to become more or less similar to normal monocytes; therefore these compounds are potential inducers of monocytic differentiation. *ATRA *is displayed on the left side of this panel and represents an effect that is more similar to granulocytic than monocytic differentiation.

Figure 4C, another enlarged portion of panel *A*, shows an area of the **Y**-distribution around the normal granulocytes (poly). In addition to the granulocytes, this area also contains duplicates of APL samples before and after several drug treatments. The effect of drugs on APL, as measured by the change of gene expression levels, is less compared to the effect of the same drugs on the HL-60 cells. The result of *Keto*- and *Phen*-treatment on APL cells is different from that of *ATRA*; while *EGFR *and *Erythro *causes only a minor change in the expression pattern of APL cells.

Figure 4D zooms in the area of the **Y**-distribution that is surrounding the undifferentiated HL-60 cells (undiff) at the third end of the **Y**. Two compounds, *5-FU*, and *5-FUD*, cause a change in the gene expression pattern that is different from monocytic and granulocytic differentiation. The *Scop*- and *Caff*-treated samples are very similar to each other and represent only a small change from the untreated HL-60 cells. The effect of two other compounds, *Apo *and *Perg *on HL-60 cells is unique and cannot be classified as only monocytic or granulocytic. At the same time, *VitD *is displayed close to the *ATRA*-treated samples in the left upper corner of panel *D*, which suggests that calcitriol's effect on HL-60 cells is similar to that of *ATRA*. This functional similarity is supported by the known similarities of action between these two compounds. Both chemicals bind to nuclear receptors, *VitD *binds to vitamin D receptors (VDR) and *ATRA *binds to retinoic acid receptors (RAR). Both of these receptors heterodimerize with retinoid X receptor (RXR) and act as hormone-dependent transcription factors.

Stegmaier *et al*. computed "monocyte and neutrophil scores" based on four parameters: *interleukin 1 receptor antagonist *(IL1RN) and *secreted phosphoprotein 1 *(SPP1) for the monocyte signature genes and *autosomal chronic granulomatous disease protein *(NCF1) and *orosomucoid 1 *(ORM1) for the neutrophil signature genes. In a supplementary table, these four values were presented for undifferentiated HL-60 cells and cells treated with one of 16 compounds. In Figure 4E, we present a two dimensional Isomap model built only from these four parameters. Arrows point to the directions of the granulocytic (left) and monocytic (right) differentiation. It is noteworthy that our unbiased model, using the expression levels of all the 18,400 transcripts and variants as input, offers a very similar result to the model using only four signature genes. In both models, the monocytic and granulocytic differentiations are revealed and the various chemical treatments cause similar changes relative to the two main dimensions. In both maps, *Caff *and *Scop *are proximal and represent only a small change compared to the undifferentiated HL-60 cells (undiff). *Perg, 5-FU*, and *5-FUD *cause more change and *Erythro, Apo, EGFR, Phen*, ATRA, and *VitD *are all mapped in similar relative locations in panel *A *and *E*. The only major difference between the two models is in the location of *Sulma *and *Methyl*. However, in both cases these two compounds are mapped close to each other. All these similarities underscore Isomap's ability to recognize treatment-related changes in gene expression patterns even when no *a priori *information is available about which genes are good predictors of a biological process or difference. Isomap can detect changes in gene expression signals in the background noise of tens of thousands of other genes.

**Figure 5:**
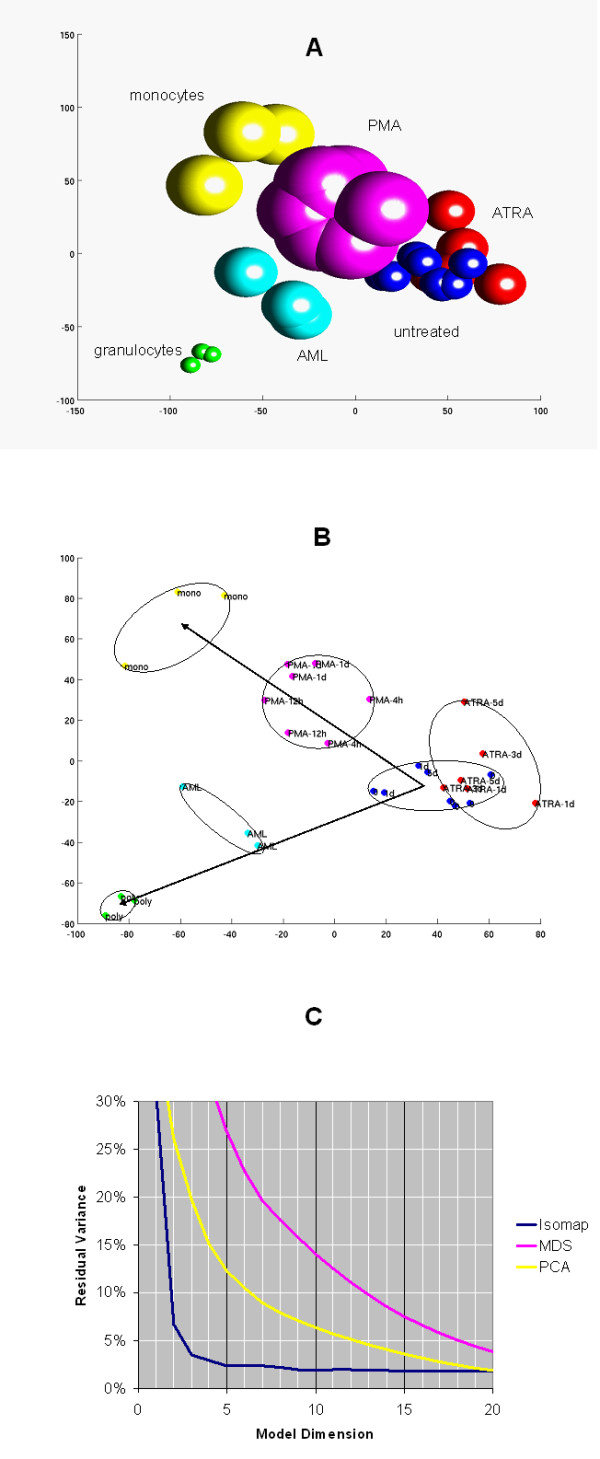
**Isomap analysis of the high throughput screening data set human 6800 arrays. **Isomap models were generated from 30 human 6800 high density oligonucleotide arrays with 7,129 genes on each array as described in *Systems and Methods*. Panel ***A ***shows a three-dimensional Isomap model. Panel ***B ***shows a two-dimensional Isomap model. Arrows point to the directions of the monocytic and granulocytic differentiation. Panel ***C ***shows the residual variance after the application of Isomap, MDS, and PCA models. Neighborhood size: k = 5. See text for more detail. Animated image is presented in Additional file 4.

Figure 5 presents the two- and three-dimensional Isomap models built from 30 Affymetrix human 6800 arrays. Panel *B *is shown to demonstrate the individual samples and the separation between sample classes. Although the sample number is relatively low, Isomap detects good separation between the patient AML, normal monocyte (mono), neutrophil (poly), *PMA*-treated, and untreated undifferentiated HL-60 sample classes. The *ATRA*-treated HL-60 class partially overlaps with the untreated HL-60 class. This is in agreement with the result of the U133A arrays that showed that *ATRA *has less effect than *PMA *on the gene expression levels in HL-60 cells. Any of these changes is less than the difference between the cell types, such as HL-60, AML, neutrophils, and monocytes. In the 6800 arrays, similar to the U133A arrays, the *PMA*-treated cells are on the monocytic differentiation axis and the AML samples are mapped on the granulocytic differentiation axis. Within the *PMA*-treated group, samples after 4 h, 12 h, and 24 h treatment are grouped together. As expected, the 4 h group is located at the right side of the *PMA *cluster closer to the untreated HL-60 cells and the 24 h group is located at the left side of the *PMA *cluster closer to the normal monocytes.

## Discussion

In this study, we use three Affymetrix high density oligonucleotide microarray data sets to demonstrate Isomap's ability to discover relevant structures in an unbiased fashion. In the rat multiple tissue gene expression data set, the structures revealed correspond to the anatomical topology of the source organs and tissues [39]. Similarly, in the high-throughput drug screening data set, the structures match the monocytic and granulocytic differentiation patterns of myeloid leukemia cells treated with various chemical compounds [40]. We also demonstrate with a complex spinal cord injury data set that Isomap reveals the three main modalities (e.g., location, time, and severity of the injury) of the experimental design [38]. This Isomap model (Fig. 2) is superior to hierarchical clustering (Fig. 1) because Isomap can model structures of higher dimensionality while hierarchical clustering is limited to the discovery of only one-dimensional structures. Clustering is limited in two significant ways; it requires well-separated data and linear correlation. The only relationship clustering can detect is a one-on-one relationship when pair-wise linear comparisons are made [41]. Isomap is a nonlinear algorithm capable of analyzing microarray data sets that are nonlinear in nature. The sources of these nonlinearities may originate from outliers, missing values, and non-normal distribution; all common events for measurements in biological systems.

In all of our examples, Isomap is applied to the expression level values of all of the genes in the microarray data set. To avoid any kind of selection bias, no filtering or data scrubbing procedures were applied. Even under these conditions, we demonstrate that Isomap is capable of finding the biologically relevant structures within noisy data sets. The experimental noise may come from the measurement error and the variations of expression levels of tens thousands of genes. When Isomap is used in production mode, the experimental noise may be decreased by eliminating the genes that are not expressed in the studied tissues and those which have very stable and unchanging expression levels in all of the samples. These procedures will further improve the resolution of the Isomap models and facilitate class discovery by Isomap. The classification power of Isomap may be improved when it is used in combination with enrichment procedures that weight the gene expression levels of different genes dependent on other surrogate information coming from the knowledge of chemical pathways in which the gene plays a role, the tissues in which the gene is expressed [42], and the gene expression patterns that are evolutionarily conserved between different organisms [39,43].

**Figure 6:**
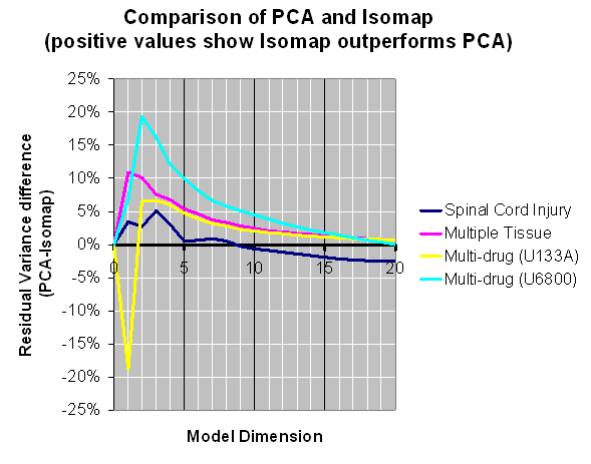
**Residual variance differences between PCA and Isomap models.**Typically, Isomap has a better performance explaining the variance in microarray datasets at lower dimensions. By increasing the number of dimensions being considered, this advantage will be eliminated.

In addition to Isomap, all the three datasets were also analyzed with PCA and MDS. Residual variance curves are presented for all the three datasets. In all instances, PCA and Isomap outperformed MDS and in most instances, Isomap worked the best out of the three methods. In the spinal cord injury dataset, PCA and Isomap give comparable results (Fig. 2D). However with 3–4-dimensional models, Isomap performs better than PCA; and at dimensions over 9, PCA worked better. In the multiple tissue dataset, Isomap outperforms PCA in the range of 2–20 dimensions (Fig. 3B). Similarly, in the two drug screening datasets, Isomap outperforms PCA at dimension 3 and over (Fig. 4F) and at every dimension (Fig. 5C). Typically, Isomap has a better performance in explaining the variance of microarray data at lower dimensions (Fig. 6). This advantage of Isomap is eliminated at higher (over 10) dimensions. The presented examples show that Isomap works well for datasets with several samples that present gradual changes from one another. However, when a few very distinct classes of samples exist then the more classical methods, e.g., PCA work better. Isomap needs samples to be present along the geodesic surface of the manifold. In our examples, many severity levels of spinal cord injury were considered, as well as multiple tissues and multiple chemical compounds, which was the source of Isomap's success. Another prerequisite for a successful application of Isomap is a large enough number of samples. Isomap will become really useful when datasets with hundreds or thousands of microarrays need to be analyzed.

The structures discovered by Isomap help to understand biological phenomena underlying these structures. Visualization tools of Isomap models may be applied as data mining tools for microarray data sets. Similar tools were used for modeling co-regulated genes in *C. elegans *with VxInsight [44] and generating high-resolution temporal models of CNS development [25]. Moreover, Isomap is a promising tool of unbiased class discovery because it is performed in a completely unsupervised manner. This is a key advantage of the Isomap algorithm considering the limited number of class discovery tools [23,45,46] compared to the abundance of class prediction methods. The Isomap algorithm is capable of revealing structures in microarray data sets, which may provide insight into underlying biological networks [4,5,7,22,25,29,30,42,43,47].

Another application of the Isomap algorithm is predicting class membership. We previously demonstrated the use of Isomap for class prediction in a breast cancer outcome microarray data set [36]. The selection of good classifiers that are robust, insensitive to outliers, medically interpretable, having high generalization power, and based on the smallest possible subset with the maximal discriminatory features [48] is important and should be evaluated for Isomap in the future. Aclass prediction model using the Isomap algorithm does not need to use all the genes in the microarray. Somorjai *et al*. argue that no matter what feature selection approach is used to the microarray data, generally 50 or more features are needed for classification [48]. In this paper, we show that only four genes are used as input for an Isomap model (Fig. 4E). Isomap is a type of dimensionality reduction method and may be evaluated as input to supervised machine learning techniques, such as ANN and SVM. Besides gene expression microarrays, Isomap can be used on other types of biological data, such as genomic, proteomic, and metabolomic data sets to reveal low dimensional structures related to diet-genome interactions, genotype-disease associations, and drug-gene-disease relationships.

## Conclusion

Isomap, a nonlinear dimensionality reduction algorithm, discovers low-dimensional structures embedded in high-dimensional Affymetrix high-density oligonucleotide microarray data sets. These structures correspond to and help to interpret underlying biological phenomena present in these data. Our work provides examples of three experiments with temporal, spatial, and functional processes revealed by the Isomap algorithm. Visualization of Isomap models helps to understand these processes and provides means of data mining from gene expression data sets. The low-dimensional models generated by Isomap help to reveal new sample classes and are potentially useful for class prediction. In summary, Isomap is a promising new algorithm for the unbiased analysis of high-density oligonucleotide microarray data sets.

## Methods

### Datasets

**Spinal cord injury data set: **A large data set consisting of 170 Affymetrix rat U34A high density oligonucleotide arrays with 8,799 genes on each array was accessed at the Gene Expression Omnibus (GEO) GSE 464. **Rat multiple tissue data set: **Another data set consisting of 122 Affymetrix rat U34A arrays with 8,799 genes on each array was accessed at the GEO GSE 952. **High throughput screening data set: **A gene expression data set based on high throughput screening (GE-HST) was accessed at the GEO GSE 995. This series is a combination of three other series: GSE 976, 982, and 985. The accessed data set contains 86 human genomic U133A Affymetrix arrays with 18,400 transcripts and variants on each array and 30 human 6800 arrays with 7,129 genes on each array.

### Probe-level microarray data analysis

All microarray data sets were downloaded to a local computer in Affymetrix CEL file format. Some of these files were text files, others were binary CEL files. Probe-level data analysis was carried out with Bioconductor 1.3.28 libraries [49] in the R 1.8.0 environment on a dual processor PC with RedHat 8.0 operating system installed. Raw data were first normalized with quantile normalization; background correction and gene expression levels were then computed with Robust Multichip Average (RMA) [50].

### Hierarchical clustering

Gene expression levels were expressed as RMA values and imported into Gene Cluster 3.0 [4]. Genes were centered to the mean and filtered with the criteria of genes with expression levels ≥ 1.0 in at least 10% of the samples. Since the RMA values are base-two logarithm-converted values, this statement is equivalent to a requirement that the expression levels of the selected genes be ≥ 2-fold or ≤ 1/2 of the mean expression level of that gene in at least 10% of the samples. In case of the spinal cord injury data set, 397 of 8,799 genes passed these filtering criteria. The selected genes and all the samples were clustered using centroid linkage hierarchical clustering based on uncentered correlation similarity metric. Branches of the clustering trees were then folded using SOM and visualized using Java TreeView 1.0.1. The result of this analysis with the spinal cord injury data set is presented in Figure 1.

### Isomap analysis of the microarray data sets

Gene expression levels of all genes were expressed as RMA values and the result table was imported into the Matlab environment. Sample-to-sample differences between all pairs of genes were expressed as Euclidean distances of RMA values. Isomap was carried out in Matlab with the algorithm provided by Joshua Tenenbaum *et al*. [35]. We used the nearest neighbor method with *k *= 2, 3, or 4 dependent on which value of *k *generated the minimal residual variance. Other data processing and visualization steps with the Isomap models were carried out with custom written Matlab functions. Source code is presented in Additional Files 5, 6, 7, 8.

### Isomap analysis of the monocytic and neutrophilic differentiation markers

Stegmaier *et al*. computed "monocyte and neutrophil scores" based on four parameters: *interleukin 1 receptor antagonist *(IL1RN) and *secreted phosphoprotein 1 *(SPP1) for the monocyte signature genes and *autosomal chronic granulomatous disease protein *(NCF1) and *orosomucoid 1 *(ORM1) for the neutrophil signature genes [40]. In a supplementary table of their paper, these four variables were presented for undifferentiated HL-60 cells as well as for cells treated with one of 16 chemical compounds. In this study, we expressed sample-to-sample differences as Euclidean distances of the base-two logarithm-converted mean values of the four marker genes. An Isomap model using the nearest neighbor method with *k *= 2 was performed with the algorithm provided by Joshua Tenenbaum [35]. All other data processing and visualization steps were carried out with custom written Matlab functions. The result of this analysis is presented in Figure 4E.

## Authors' contributions

KD conceived of and designed the study, carried out the data analysis and visualization, developed the Matlab computer code, and drafted the manuscript. RLR and WM contributed to the study design and editing of the manuscript. All authors read and approved the final manuscript.

## Supplementary Material

Additional File 5 **Matlab code**. Gene expression values are stored in a matrix (M) with variables (genes) in the rows and observations (samples) in the columns. A vector (samples) contains the sample names. Another vector (pheno) contains the classification of each sample. The gene names are stored in another vector (genes). Euclidean distances may be computed as D = L2_distance(M, M); If we want to perform Isomap at dimensions 1 through 20 then we store this range in: options.dims = 1:20; The Isomap algorithm can be executed with a neighborhood size of 5 as follows: [Y, R] = Isomap(D,'k',5, options); 1 through 20 dimensional coordinates are stored in Y. coords and the residual variances are stored in R. The results can be wrapped around to create a 3D 'model' structure, using makemodel: model = makemodel(M, Y, options, samples, pheno,'i',3,5); Click here for file

Additional File 6 **Matlab code**. The function showmode can be used l to render an image from this model: showmodel(model); Enter showmodel without input parameters for more options. Click here for file

Additional File 7 **Matlab code**. Adding the sample names to the figure: annotate(Y, samples,3); Click here for file

Additional File 8 **Matlab code**. Similarly, a 3-D animation (mov) can be created using: mov = makemovie(model); Enter makemovie without input parameters for more options. Click here for file

Additional File 1 **Animated Isomap model of **Fig. 2A. Click here for file

Additional File 2 **Animated Isomap model of **Fig. 2B. Click here for file

Additional File 3 **Animated Isomap model of **Fig. 2C. Click here for file

Additional File 4 **Animated Isomap model of **Fig. 5A. Click here for file
