# Supplementary figures and images for: Sample phenotype clusters in high-density oligonucleotide microarray data sets are revealed using Isomap, a nonlinear algorithm
Source: BMC Bioinformatics. 2005 Aug 2;6:195. doi: 10.1186/1471-2105-6-195 (PMC1189082; doi:10.1186/1471-2105-6-195)

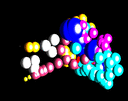

Supplement: Additional File 1 — Animated Isomap model of Fig. 2A. [file 1471-2105-6-195-S1.gif]

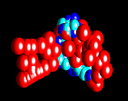

Supplement: Additional File 2 — Animated Isomap model of Fig. 2B. [file 1471-2105-6-195-S2.gif]

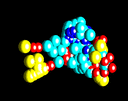

Supplement: Additional File 3 — Animated Isomap model of Fig. 2C. [file 1471-2105-6-195-S3.gif]

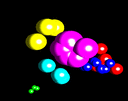

Supplement: Additional File 4 — Animated Isomap model of Fig. 5A. [file 1471-2105-6-195-S4.gif]
